# Hypertrophied reversed palmaris longus muscle (pseudotumor) of the forearm causing median nerve compression: a case report

**DOI:** 10.1186/s13256-020-02368-y

**Published:** 2020-05-25

**Authors:** Majdi Hashem, Raheef Alatassi, Kaushal Narinder, Fawaz Emran

**Affiliations:** 1 Department of Orthopedic Surgery, Imam Mohammad Ibn Saud Islamic University, College of Medicine, 7544, Riyadh, 11432 Saudi Arabia; 2 Department of Orthopedic Surgery, McGill University Health Centre, Jewish General Hospital, 3755 Côte Ste-Catherine Road, Montreal, H3T 1E2 Canada; 3 Department of Orthopedic Surgery, Security Forces Hospital, P.O. Box: 3643, Riyadh, 11481 Saudi Arabia; 4 Department of Radiology, Prince Faisal Bin Fahad Bin Abdulziz Sports Medicine Hospital, 2306, Riyadh, 12752 Saudi Arabia; 5 Department of Surgery, Family Care Hospital, 7859, Riyadh, 13213 Saudi Arabia

**Keywords:** Palmaris longus, Muscle hypertrophy, Muscle anomaly, Muscle variant, Magnetic resonance image, Median nerve compression

## Abstract

**Background:**

The palmaris longus muscle is considered one of the most anatomically variable muscles in the human body. Localized swelling of the forearm due to hypertrophy of the palmaris longus muscle is rare.

**Case presentation:**

Here, we report a rare case of a 24-year-old Arab man who presented with a painful mass on his forearm with symptoms of median nerve compression. A full radiological assessment was performed, and he was treated conservatively.

**Conclusion:**

This case confirmed that a hypertrophied palmaris longus muscle can be the cause of swelling on the forearm and should always be considered in the differential diagnosis. With this report, we aimed to increase awareness regarding the unusual variations of palmaris longus muscle and the importance of using radiological investigations to establish a diagnosis.

## Introduction

Swelling on the forearm is usually because of a trauma, such as fracture, tendonitis, or muscle tear. It could also be due to inflammatory conditions like cellulitis, abscess, osteomyelitis, or phlebitis. Non-traumatic causes of swelling on the forearm are generally uncommon. Recently, several authors have described different variations in the morphology, agenesis, and location of the palmaris longus muscle (PLM) [1–3]. Localized swelling of the forearm due to hypertrophy of the PLM is very rare [4–7]. As it is unusual to see such cases, diagnosing hypertrophy of the PLM as the cause of a swelling can be difficult and confusing.

Here we report a rare case of an unusual variation of the PLM that presented as a painful swelling on the distal forearm. In this case, a radiological approach was used to diagnose the cause of the swelling. This approach was effective for this case and can help diagnose similar cases in the future.

## Case presentation

A 24-year-old right-handed Arab man, working as a computer engineer, presented with a painful mass on his right wrist that had appeared 2 years ago. The mass had slowly grown over the past 2 years and the pain had increased over time. It started affecting the range of motion of his right wrist, specifically during flexion, but without a significant effect on the range of motion of his fingers. He described experiencing a tingling sensation at the tip of the second and third fingers of his right hand. The mass started to affect his daily activity, especially at work while using a keyboard for long periods. He had no history of trauma to his right hand or of any chronic medical illnesses.

**Fig. 1:**
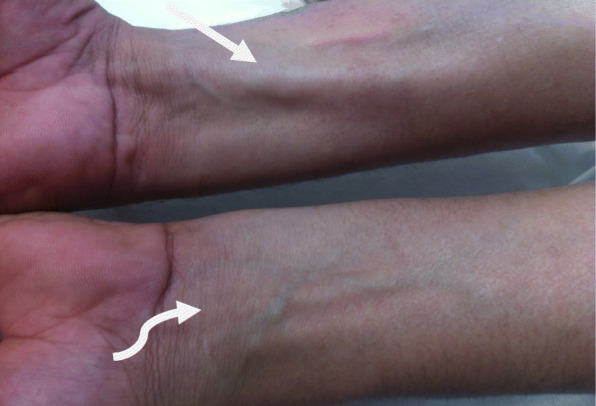
Photograph obtained during the initial examination of the patient. An anterior view showing abnormal thickening of the muscle in the right forearm (the *straight arrow* indicates the hypertrophied muscle of the right forearm and the *curved arrow* indicates the normal muscle on the other hand)

He initially visited a local clinic, where he was diagnosed as having a lipoma-like tumor on his right forearm. He was referred to our hospital for further evaluation. On examination, we noted an ill-defined firm fusiform mass along the volar aspect of his distal forearm, approximately 3-cm proximal to his wrist joint. The mass was approximately 2 × 3 × 3 in volume and was not pulsatile. It became more prominent during flexion of the wrist (Fig. 1). Moreover, there was no focal tenderness or changes in the skin covering the mass.

**Fig. 2:**
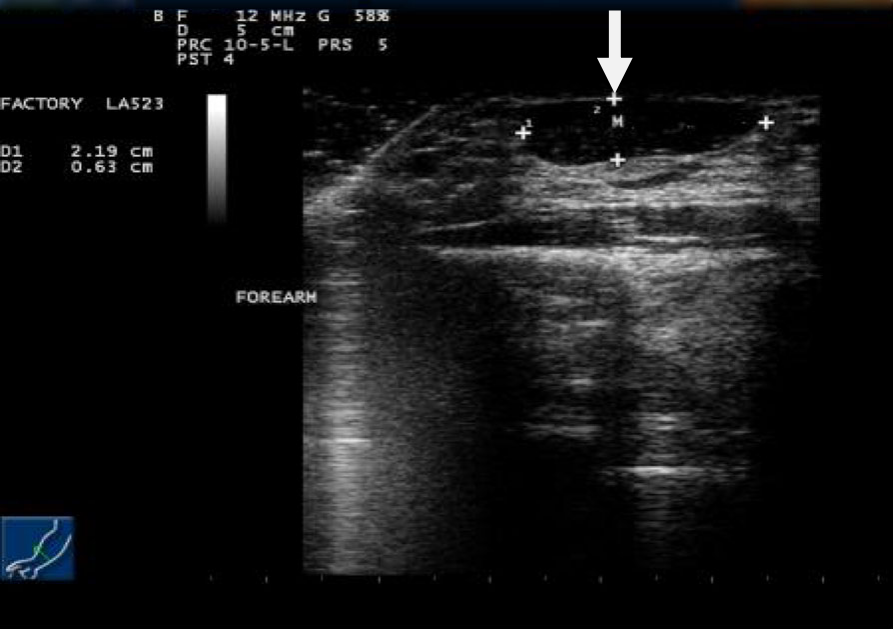
Ultrasonographic image of the right forearm in transverse view revealing the muscle mass (the *arrow* indicates the hypertrophied muscle)

**Fig. 3:**
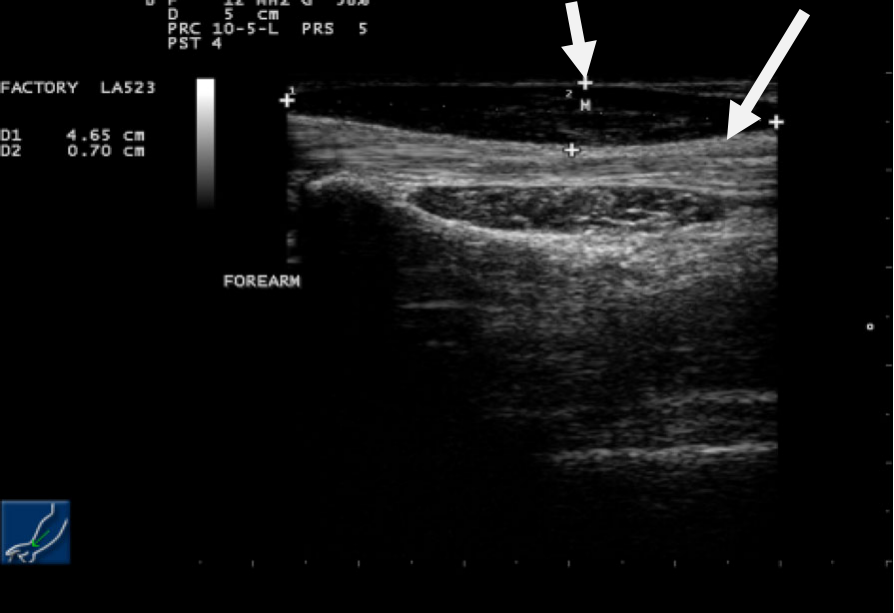
Ultrasonographic image of the right forearm in longitudinal view revealing the muscle mass (the *small arrow* indicates the hypertrophied muscle and the *large arrow* indicates the median nerve)

**Fig. 4:**
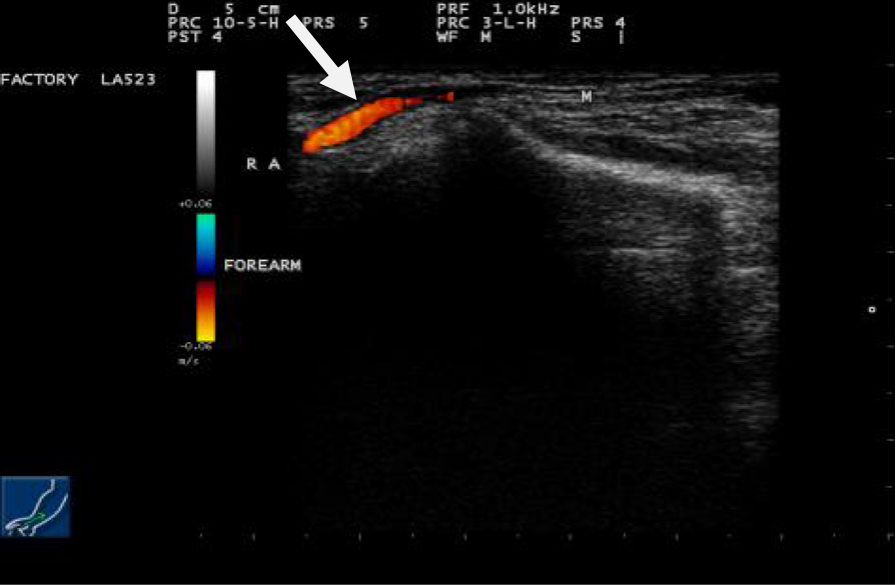
Doppler ultrasonographic image of the right forearm in longitudinal view revealing the muscle mass completely separated from the radial artery (the *arrow* indicates the radial artery)

Simple radiography of his forearm performed initially revealed no bony abnormalities. Ultrasonography showed a superficial soft tissue mass at the level of the subcutaneous plane. It was isoechoic to the muscles of the forearm (Figs. 2 and 3). The mass was clearly observed in Doppler imaging and did not show any cystic changes or abnormal vascularity (Fig. 4).

**Fig. 5:**
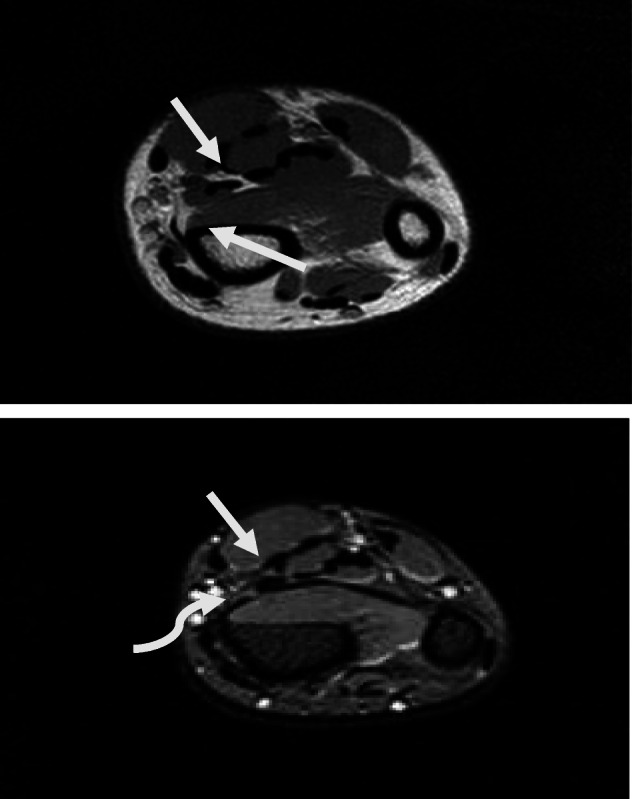
**a** T1 and **b** T2 fast field echo magnetic resonance imaging of the right distal forearm (axial scan) revealing that the mass is isointense with the muscles of the forearm (the *straight* arrow indicates the hypertrophied muscle and the *curved arrow* indicates the median nerve)

Magnetic resonance imaging of his right forearm was performed. T1-weighted images and T2-weighted images in the axial, sagittal, and coronal positions revealed signal intensities of the mass similar to those of the adjacent muscles (Fig. 5). Thinning of the superficial fascia and subcutaneous fat was also observed.

**Fig. 6:**
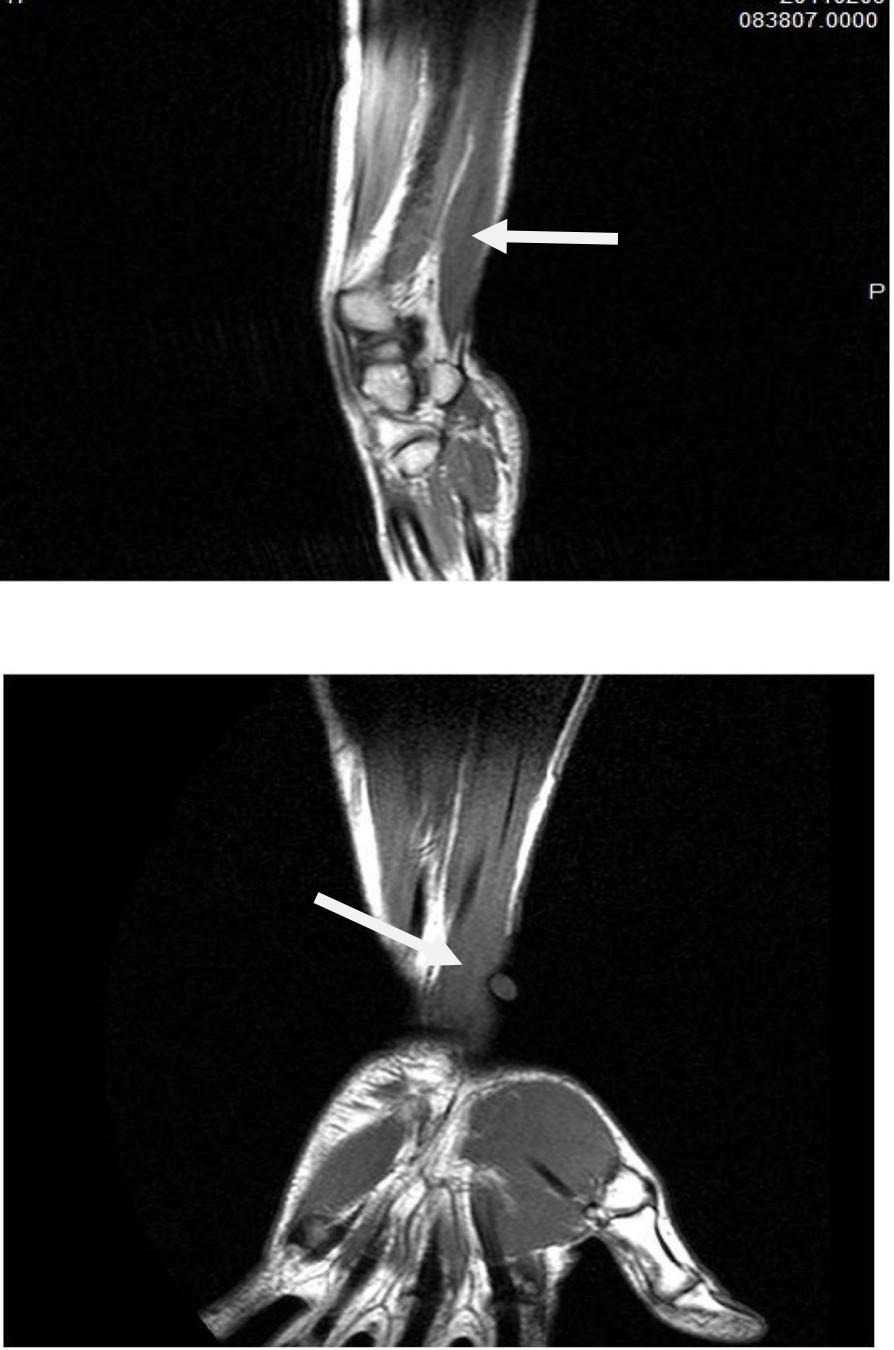
T1-weighted (**a**) sagittal and (**b**) coronal magnetic resonance images of the right distal forearm showing the mass isointense with the muscles of the forearm (the *arrow* indicates the hypertrophied muscle)

The mass was located medial to the tendon of the flexor carpi radialis and in the region of the tendon of palmaris longus. The muscle appeared tendinous in the upper (proximal) portion and muscular in its lower (distal) portion. The sagittal plane images showed the longitudinal fusiform extent of the mass, which reached the upper margin of the flexor retinaculum at its distal extent (Fig. 6).

The mass was located close to the median nerve, as observed in the axial images. We diagnosed our patient as having reversed PLM. We detected marked hypertrophy of the belly of the muscle extending almost up to its point of attachment with the flexor retinaculum and restricted movement of its tendon over the flexor retinaculum. In addition, the median nerve near the wrist joint was being compressed. The pressure on the median nerve was the most probable cause of the wrist pain experienced by our patient.

We planned to treat our patient conservatively. A systemic anti-inflammatory medication and a wrist splint was the first line of treatment with regular follow-up. Furthermore, we referred him to a physiotherapy department to start stretching exercises. During his follow-up visits, he was able to tolerate pain. One year later, he eventually regained full function of his right hand, and it no longer affected his daily activities.

## Discussion and conclusions

Our patient presented with a painful swelling of the distal forearm and median nerve compression symptoms. In such cases, where no traumatic or inflammatory causes are present for the swelling but the mass continues growing gradually, other rarer causes must be considered.

Full radiological investigations help establish the diagnosis in such cases. Initially, simple radiography should be performed to rule out the presence of any bony deformities, including fractures, enthesophytes, and bony tumors. If radiography gives a negative result, ultrasonography must be performed before proceeding to more advanced imaging. In ultrasonography, a mass that is isoechoic to the surrounding muscles will be highly suggestive of a muscle anomaly. PLM has many anatomical variations as has been described in the existing literature. These variations include reversed, duplicated, bifid, hypertrophied, or complete absence of PLM [1–3]. Reversed, bifid, and hypertrophied PLM may present as a pseudomass and all of them will appear isoechoic in an ultrasonographic image [3]. The next step to distinguish between these conditions is to perform magnetic resonance imaging. Using T1-weighted imaging with a high-resolution surface coil and a 256 × 256 matrix will be highly accurate in ruling out the existence of a tumor. In addition, it will show the exact muscle orientation and its origin and insertion [7].

Hypertrophied PLM can cause compression to the near neurovascular structure through an increase in its size. A Doppler ultrasonography must be performed to evaluate the adjacent vessels and to ensure that the muscle mass is not compressing them.

For symptomatic masses, conservative management should be used to treat the most painful cases; surgery is usually not necessary. Systemic, anti-inflammatory medications, activity modification with stretching exercises, and wrist splint all are considered conservative management [8, 9]. Although surgical intervention is not preferable as it can lead to complications, it is indicated when all conservative measures have failed.

We present here a rare case of an unusual swelling that stemmed from hypertrophied PLM in the forearm of a healthy 24-year-old man, which was treated conservatively and with satisfactory improvement of symptoms. Very few similar cases have been reported in the literature.

To conclude, this case confirmed that a hypertrophied PLM can be the cause of swelling on the forearm with median nerve compression and should always be considered in the differential diagnosis. This report aimed to increase the awareness of unusual variations of PLM and the importance of using radiological investigation to obtain a diagnosis.

## Data Availability

The datasets used and/or analyzed during the current study are available from the corresponding author on reasonable request.
